# 3-(3-Chloro­benzo­yl)-4-hydr­oxy-2*H*-1,2-benzothia­zine 1,1-dioxide

**DOI:** 10.1107/S1600536810009761

**Published:** 2010-03-20

**Authors:** Zunera Khalid, Hamid Latif Siddiqui, Matloob Ahmad, Sana Aslam, Masood Parvez

**Affiliations:** a Institute of Chemistry, University of the Punjab, Lahore 54590, Pakistan; b Applied Chemistry Research Centre, PCSIR Laboratories Complex, Lahore 54600, Pakistan; c Department of Chemistry, The University of Calgary, 2500 University Drive NW, Calgary, Alberta, Canada T2N 1N4

## Abstract

In the title compound, C_15_H_10_ClNO_4_S, the heterocyclic thia­zine ring adopts a half-chair conformation with the S and N atoms displaced by 0.476 (5) and 0.227 (5) Å, respectively, on opposite sides of the mean plane formed by the remaining ring atoms. The structure is stabilized by inter­molecular N—H⋯O and C—H⋯O hydrogen bonds. In addition, intra­molecular O—H⋯O and C—H⋯N inter­actions are also present.

## Related literature

For the biological activity of 1,2-benzothia­zine derivatives, see: Ahmad *et al.* (2010 ▶); Lombardino & Wiseman, (1972 ▶); Gupta *et al.* (1993 ▶, 2002 ▶); Zia-ur-Rehman *et al.* (2006 ▶); Berryman *et al.* (1998 ▶). For comparative bond distances, see: Allen *et al.* (1987 ▶). For related structures, see: Siddiqui *et al.* (2008 ▶)
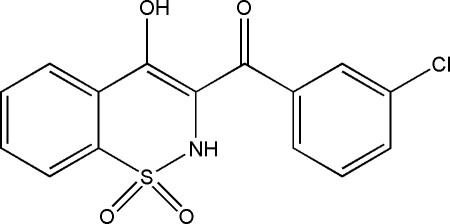


## Experimental

### 

#### Crystal data

C_15_H_10_ClNO_4_S
*M* *_r_* = 335.75
Triclinic, 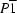

*a* = 4.7151 (3) Å
*b* = 12.2879 (8) Å
*c* = 12.5809 (6) Å
α = 81.375 (3)°
β = 84.463 (3)°
γ = 85.608 (3)°
*V* = 715.88 (7) Å^3^
*Z* = 2
Mo *K*α radiation
μ = 0.43 mm^−1^
*T* = 295 K
0.14 × 0.12 × 0.10 mm

#### Data collection

Nonius KappaCCD diffractometer
Absorption correction: multi-scan (*SORTAV*; Blessing, 1997 ▶) *T*_min_ = 0.942, *T*_max_ = 0.958
4352 measured reflections
3202 independent reflections
2783 reflections with *I* > 2σ(*I*)
*R*_int_ = 0.027

#### Refinement

*R*[*F* ^2^ > 2σ(*F* ^2^)] = 0.051
*wR*(*F* ^2^) = 0.121
*S* = 1.09
3202 reflections
200 parameters
H-atom parameters constrained
Δρ_max_ = 0.45 e Å^−3^
Δρ_min_ = −0.36 e Å^−3^

### 

Data collection: *COLLECT* (Hooft, 1998 ▶); cell refinement: *DENZO* (Otwinowski & Minor, 1997 ▶); data reduction: *SCALEPACK* (Otwinowski & Minor, 1997 ▶); program(s) used to solve structure: *SHELXS97* (Sheldrick, 2008 ▶); program(s) used to refine structure: *SHELXL97* (Sheldrick, 2008 ▶); molecular graphics: *ORTEP-3 for Windows* (Farrugia, 1997 ▶); software used to prepare material for publication: *SHELXL97*.

## Supplementary Material

Crystal structure: contains datablocks global, I. DOI: 10.1107/S1600536810009761/pk2231sup1.cif

Structure factors: contains datablocks I. DOI: 10.1107/S1600536810009761/pk2231Isup2.hkl

Additional supplementary materials:  crystallographic information; 3D view; checkCIF report

## Figures and Tables

**Table 1:** Hydrogen-bond geometry (Å, °)

*D*—H⋯*A*	*D*—H	H⋯*A*	*D*⋯*A*	*D*—H⋯*A*
N1—H1*N*⋯O2^i^	0.86	2.03	2.872 (3)	168
O3—H3*O*⋯O4	0.82	1.80	2.525 (3)	146
C2—H2⋯O1^ii^	0.93	2.54	3.279 (3)	136
C14—H14⋯O2^iii^	0.93	2.58	3.435 (3)	153
C15—H15⋯N1	0.93	2.54	3.009 (4)	112

Symmetry codes: (i) 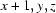; (ii) 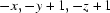; (iii) 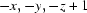.

**Fig. 1.**
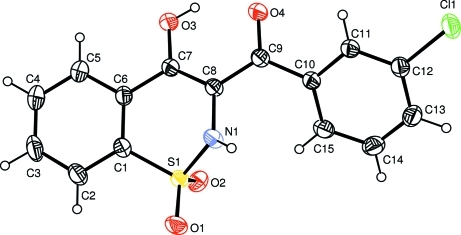
The title molecule with the displacement ellipsoids plotted at 30% probability level (Farrugia, 1997).

## supplementary crystallographic information

### Comment

1,2-Benzothiazine 1,1-dioxides represent a class of pharmaceutically important heterocyclic compounds that have received considerable attention because of their dynamic structural features and a wide range of biological activity, e.g., anti-inflammatory (Lombardino & Wiseman, 1972), analgesic (Gupta *et al.*, 2002), anti-cancer (Gupta *et al.*, 1993), anti-bacterial (Zia-ur-Rehman *et al.*, 2006) and endothelin receptor antagonists (Berryman *et al.*, 1998), etc. In continuation of our research on the synthesis of biologically active benzothiazine derivatives (Ahmad *et al.*, 2010), we herein report the synthesis and crystal structure of the title compound.

The title molecule is presented in Fig. 1. The bond distances are as expected (Allen *et al.*, 1987) and agree with the corresponding parameters reported in closely related compounds (Siddiqui *et al.*, 2008). The heterocyclic thiazine ring adopts a half chair conformation with atoms S1 and N1 displaced by 0.476 (5) and 0.227 (5) Å , respectively, on the opposite sides from the mean plane formed by the remaining ring atoms.

The structure is stabilized by intermolecular hydrogen bonds of the types N—H···O and C—H···O. In addition, intramolecular interactions O3—H3O···O4 and C15—H15···N1 are also present consolidating the crystal packing; details are provided in Table 1.

### Experimental

Sodium metal (4.83 g, 210 mmol) was dissolved in dry methanol (35 ml) and 2-[2-(3-chlorophenyl)-2-oxoethyl]-1,2-benzisothiazol-3(2*H*)-one 1,1-dioxide (10.07 g, 30 mmol) was added to it. The mixture was refluxed for 30 minutes. The contents of the flask were cooled to room temperature and pH was adjusted at 3.0 using 5% HCl. A pale yellow precipitate of the title compound was filtered and washed with cold methanol. Crystals suitable for crystallographic study were grown from a methanolic solution by slow evaporation at room temperature. Yield, 74%; m.p. 438-440 K.

### Refinement

Though all the H atoms could be distinguished in the difference Fourier map, they were included at geometrically idealized positions and refined using a riding-model approximation with the following constraints: O—H, N—H and C—H distances were set to 0.82, 0.86 and 0.93 Å, respectively, and *U*_iso_(H) = 1.2*U*_eq_(parent atom). The final difference map was essentially featureless.

### Crystal data

**Table:**

C_15_H_10_ClNO_4_S	*Z* = 2
*M**_r_* = 335.75	*F*(000) = 344
Triclinic, *P*1	*D*_x_ = 1.558 Mg m^−^^3^
Hall symbol: -P 1	Mo *K*α radiation, λ = 0.71073 Å
*a* = 4.7151 (3) Å	Cell parameters from 1649 reflections
*b* = 12.2879 (8) Å	θ = 1.0–27.5°
*c* = 12.5809 (6) Å	µ = 0.43 mm^−^^1^
α = 81.375 (3)°	*T* = 295 K
β = 84.463 (3)°	Block, yellow
γ = 85.608 (3)°	0.14 × 0.12 × 0.10 mm
*V* = 715.88 (7) Å^3^	

### Data collection

**Table:**

Nonius KappaCCD diffractometer	3202 independent reflections
Radiation source: fine-focus sealed tube	2783 reflections with *I* > 2σ(*I*)
graphite	*R*_int_ = 0.027
ω and φ scans	θ_max_ = 27.5°, θ_min_ = 1.6°
Absorption correction: multi-scan (*SORTAV*; Blessing, 1997)	*h* = −6→6
*T*_min_ = 0.942, *T*_max_ = 0.958	*k* = −15→15
4352 measured reflections	*l* = −16→16

### Refinement

**Table:**

Refinement on *F*^2^	Primary atom site location: structure-invariant direct methods
Least-squares matrix: full	Secondary atom site location: difference Fourier map
*R*[*F*^2^ > 2σ(*F*^2^)] = 0.051	Hydrogen site location: difference Fourier map
*wR*(*F*^2^) = 0.121	H-atom parameters constrained
*S* = 1.09	*w* = 1/[σ^2^(*F*_o_^2^) + (0.025*P*)^2^ + 0.745*P*] where *P* = (*F*_o_^2^ + 2*F*_c_^2^)/3
3202 reflections	(Δ/σ)_max_ < 0.001
200 parameters	Δρ_max_ = 0.45 e Å^−^^3^
0 restraints	Δρ_min_ = −0.36 e Å^−^^3^

### Special details

**Table:**

Experimental. IR (KBr) 3157, 1615, 1358, 1156 cm^-1^, MS m/z: 335.2 [M^+^]. ^1^H NMR (DMSO-d_6_); 7.64 (t, 2H, J = 8.0 Hz, Ar—H), 7.75 (d, 2H, J = 8.0 Hz, Ar—H), 7.96 (d, 1H, J = 10.0 Hz, Ar—H), 7.96 (s, 1H, J = 16.4 Hz, Ar—H), 8.18 (t, 2H, J = 3.2 Hz, Ar—H).
Geometry. All esds (except the esd in the dihedral angle between two l.s. planes) are estimated using the full covariance matrix. The cell esds are taken into account individually in the estimation of esds in distances, angles and torsion angles; correlations between esds in cell parameters are only used when they are defined by crystal symmetry. An approximate (isotropic) treatment of cell esds is used for estimating esds involving l.s. planes.
Refinement. Refinement of *F*^2^ against ALL reflections. The weighted *R*-factor wR and goodness of fit *S* are based on *F*^2^, conventional *R*-factors *R* are based on *F*, with *F* set to zero for negative *F*^2^. The threshold expression of *F*^2^ > σ(*F*^2^) is used only for calculating *R*-factors(gt) etc. and is not relevant to the choice of reflections for refinement. *R*-factors based on *F*^2^ are statistically about twice as large as those based on *F*, and *R*- factors based on ALL data will be even larger.

### Fractional atomic coordinates and isotropic or equivalent isotropic displacement parameters (Å^2^)

**Table:**

	*x*	*y*	*z*	*U*_iso_*/*U*_eq_	
Cl1	1.12832 (17)	−0.15369 (6)	0.14547 (7)	0.0618 (2)	
S1	−0.00971 (13)	0.31098 (5)	0.38523 (5)	0.03988 (17)	
O1	0.1096 (5)	0.34309 (18)	0.47468 (16)	0.0601 (6)	
O2	−0.2260 (4)	0.23281 (16)	0.40650 (15)	0.0511 (5)	
O3	−0.0663 (5)	0.30935 (17)	0.04835 (15)	0.0604 (6)	
H3O	0.0372	0.2596	0.0257	0.073*	
O4	0.3079 (4)	0.15059 (17)	0.05644 (14)	0.0553 (5)	
N1	0.2456 (4)	0.26558 (18)	0.30655 (17)	0.0441 (5)	
H1N	0.4144	0.2519	0.3277	0.053*	
C1	−0.1507 (5)	0.4240 (2)	0.3008 (2)	0.0415 (5)	
C2	−0.2846 (7)	0.5148 (2)	0.3434 (3)	0.0563 (7)	
H2	−0.2815	0.5204	0.4162	0.068*	
C3	−0.4223 (8)	0.5961 (3)	0.2754 (3)	0.0715 (10)	
H3	−0.5125	0.6575	0.3025	0.086*	
C4	−0.4271 (9)	0.5873 (3)	0.1678 (3)	0.0748 (10)	
H4	−0.5241	0.6421	0.1233	0.090*	
C5	−0.2904 (7)	0.4985 (3)	0.1253 (3)	0.0623 (8)	
H5	−0.2926	0.4943	0.0522	0.075*	
C6	−0.1488 (5)	0.4148 (2)	0.1915 (2)	0.0425 (5)	
C7	−0.0006 (5)	0.3206 (2)	0.1462 (2)	0.0415 (5)	
C8	0.1900 (5)	0.2482 (2)	0.20148 (19)	0.0386 (5)	
C9	0.3263 (5)	0.1547 (2)	0.1542 (2)	0.0407 (5)	
C10	0.4876 (5)	0.0619 (2)	0.21729 (19)	0.0394 (5)	
C11	0.7101 (5)	0.0062 (2)	0.1617 (2)	0.0413 (5)	
H11	0.7634	0.0309	0.0895	0.050*	
C12	0.8495 (5)	−0.0854 (2)	0.2149 (2)	0.0435 (6)	
C13	0.7712 (7)	−0.1251 (2)	0.3214 (2)	0.0553 (7)	
H13	0.8680	−0.1870	0.3562	0.066*	
C14	0.5477 (7)	−0.0714 (2)	0.3752 (2)	0.0589 (8)	
H14	0.4910	−0.0984	0.4465	0.071*	
C15	0.4057 (6)	0.0222 (2)	0.3248 (2)	0.0494 (6)	
H15	0.2568	0.0583	0.3623	0.059*	

### Atomic displacement parameters (Å^2^)

**Table:**

	*U*^11^	*U*^22^	*U*^33^	*U*^12^	*U*^13^	*U*^23^
Cl1	0.0599 (4)	0.0588 (4)	0.0699 (5)	0.0225 (3)	−0.0186 (4)	−0.0246 (4)
S1	0.0367 (3)	0.0471 (3)	0.0379 (3)	0.0050 (2)	−0.0090 (2)	−0.0131 (2)
O1	0.0647 (13)	0.0721 (14)	0.0503 (11)	0.0158 (10)	−0.0234 (10)	−0.0297 (10)
O2	0.0417 (10)	0.0556 (11)	0.0526 (11)	−0.0028 (8)	−0.0053 (8)	0.0035 (9)
O3	0.0839 (15)	0.0576 (13)	0.0399 (10)	0.0222 (11)	−0.0180 (10)	−0.0126 (9)
O4	0.0659 (13)	0.0607 (12)	0.0395 (10)	0.0191 (10)	−0.0104 (9)	−0.0160 (8)
N1	0.0318 (10)	0.0567 (13)	0.0484 (12)	0.0085 (9)	−0.0125 (9)	−0.0226 (10)
C1	0.0394 (13)	0.0387 (12)	0.0480 (14)	0.0002 (10)	−0.0061 (10)	−0.0116 (10)
C2	0.0612 (18)	0.0490 (16)	0.0619 (17)	0.0089 (13)	−0.0079 (14)	−0.0232 (13)
C3	0.084 (2)	0.0411 (16)	0.089 (3)	0.0195 (15)	−0.0117 (19)	−0.0194 (15)
C4	0.095 (3)	0.0494 (18)	0.075 (2)	0.0278 (17)	−0.0176 (19)	−0.0025 (15)
C5	0.078 (2)	0.0501 (17)	0.0551 (17)	0.0170 (15)	−0.0116 (15)	−0.0028 (13)
C6	0.0442 (13)	0.0365 (12)	0.0466 (14)	0.0033 (10)	−0.0058 (11)	−0.0067 (10)
C7	0.0455 (13)	0.0409 (13)	0.0389 (12)	0.0028 (10)	−0.0068 (10)	−0.0092 (10)
C8	0.0358 (12)	0.0424 (13)	0.0390 (12)	0.0015 (10)	−0.0045 (9)	−0.0118 (10)
C9	0.0389 (12)	0.0435 (13)	0.0409 (13)	0.0001 (10)	−0.0036 (10)	−0.0109 (10)
C10	0.0428 (13)	0.0381 (12)	0.0385 (12)	−0.0001 (10)	−0.0051 (10)	−0.0094 (9)
C11	0.0445 (13)	0.0420 (13)	0.0387 (12)	0.0008 (10)	−0.0066 (10)	−0.0099 (10)
C12	0.0437 (13)	0.0426 (13)	0.0476 (14)	0.0028 (10)	−0.0130 (11)	−0.0140 (11)
C13	0.073 (2)	0.0411 (14)	0.0527 (16)	0.0030 (13)	−0.0202 (14)	−0.0049 (12)
C14	0.082 (2)	0.0514 (16)	0.0420 (15)	−0.0081 (15)	−0.0055 (14)	−0.0009 (12)
C15	0.0567 (16)	0.0492 (15)	0.0428 (14)	−0.0057 (12)	0.0035 (12)	−0.0122 (11)

### Geometric parameters (Å, °)

**Table:**

Cl1—C12	1.739 (3)	C4—H4	0.9300
S1—O1	1.4240 (18)	C5—C6	1.394 (4)
S1—O2	1.434 (2)	C5—H5	0.9300
S1—N1	1.604 (2)	C6—C7	1.467 (3)
S1—C1	1.747 (3)	C7—C8	1.377 (3)
O3—C7	1.327 (3)	C8—C9	1.451 (3)
O3—H3O	0.8200	C9—C10	1.491 (3)
O4—C9	1.250 (3)	C10—C15	1.395 (4)
N1—C8	1.422 (3)	C10—C11	1.396 (3)
N1—H1N	0.8600	C11—C12	1.376 (3)
C1—C2	1.391 (4)	C11—H11	0.9300
C1—C6	1.396 (3)	C12—C13	1.380 (4)
C2—C3	1.380 (4)	C13—C14	1.376 (4)
C2—H2	0.9300	C13—H13	0.9300
C3—C4	1.377 (5)	C14—C15	1.385 (4)
C3—H3	0.9300	C14—H14	0.9300
C4—C5	1.376 (4)	C15—H15	0.9300
			
O1—S1—O2	118.25 (13)	O3—C7—C8	122.4 (2)
O1—S1—N1	108.39 (12)	O3—C7—C6	115.1 (2)
O2—S1—N1	109.12 (12)	C8—C7—C6	122.6 (2)
O1—S1—C1	112.18 (12)	C7—C8—N1	118.7 (2)
O2—S1—C1	106.33 (11)	C7—C8—C9	120.5 (2)
N1—S1—C1	101.20 (12)	N1—C8—C9	120.8 (2)
C7—O3—H3O	109.5	O4—C9—C8	119.2 (2)
C8—N1—S1	119.34 (16)	O4—C9—C10	117.9 (2)
C8—N1—H1N	120.3	C8—C9—C10	122.9 (2)
S1—N1—H1N	120.3	C15—C10—C11	119.6 (2)
C2—C1—C6	121.6 (2)	C15—C10—C9	122.6 (2)
C2—C1—S1	120.7 (2)	C11—C10—C9	117.4 (2)
C6—C1—S1	117.43 (18)	C12—C11—C10	119.3 (2)
C3—C2—C1	118.6 (3)	C12—C11—H11	120.4
C3—C2—H2	120.7	C10—C11—H11	120.4
C1—C2—H2	120.7	C11—C12—C13	121.6 (2)
C4—C3—C2	120.6 (3)	C11—C12—Cl1	119.0 (2)
C4—C3—H3	119.7	C13—C12—Cl1	119.3 (2)
C2—C3—H3	119.7	C14—C13—C12	118.9 (3)
C5—C4—C3	120.9 (3)	C14—C13—H13	120.5
C5—C4—H4	119.6	C12—C13—H13	120.5
C3—C4—H4	119.6	C13—C14—C15	121.0 (3)
C4—C5—C6	120.2 (3)	C13—C14—H14	119.5
C4—C5—H5	119.9	C15—C14—H14	119.5
C6—C5—H5	119.9	C14—C15—C10	119.6 (3)
C5—C6—C1	118.2 (2)	C14—C15—H15	120.2
C5—C6—C7	120.3 (2)	C10—C15—H15	120.2
C1—C6—C7	121.5 (2)		
			
O1—S1—N1—C8	−167.9 (2)	O3—C7—C8—N1	−179.3 (2)
O2—S1—N1—C8	62.0 (2)	C6—C7—C8—N1	−0.1 (4)
C1—S1—N1—C8	−49.8 (2)	O3—C7—C8—C9	−0.6 (4)
O1—S1—C1—C2	−35.9 (3)	C6—C7—C8—C9	178.6 (2)
O2—S1—C1—C2	94.9 (2)	S1—N1—C8—C7	36.5 (3)
N1—S1—C1—C2	−151.2 (2)	S1—N1—C8—C9	−142.2 (2)
O1—S1—C1—C6	150.4 (2)	C7—C8—C9—O4	12.1 (4)
O2—S1—C1—C6	−78.9 (2)	N1—C8—C9—O4	−169.2 (2)
N1—S1—C1—C6	35.0 (2)	C7—C8—C9—C10	−167.5 (2)
C6—C1—C2—C3	1.1 (5)	N1—C8—C9—C10	11.2 (4)
S1—C1—C2—C3	−172.4 (3)	O4—C9—C10—C15	−143.7 (3)
C1—C2—C3—C4	0.2 (5)	C8—C9—C10—C15	35.9 (4)
C2—C3—C4—C5	−1.3 (6)	O4—C9—C10—C11	29.3 (3)
C3—C4—C5—C6	1.2 (6)	C8—C9—C10—C11	−151.1 (2)
C4—C5—C6—C1	0.1 (5)	C15—C10—C11—C12	−1.6 (4)
C4—C5—C6—C7	−179.4 (3)	C9—C10—C11—C12	−174.8 (2)
C2—C1—C6—C5	−1.2 (4)	C10—C11—C12—C13	1.3 (4)
S1—C1—C6—C5	172.5 (2)	C10—C11—C12—Cl1	−179.47 (18)
C2—C1—C6—C7	178.3 (3)	C11—C12—C13—C14	0.2 (4)
S1—C1—C6—C7	−8.0 (3)	Cl1—C12—C13—C14	−179.1 (2)
C5—C6—C7—O3	−14.6 (4)	C12—C13—C14—C15	−1.3 (5)
C1—C6—C7—O3	165.9 (2)	C13—C14—C15—C10	0.9 (4)
C5—C6—C7—C8	166.2 (3)	C11—C10—C15—C14	0.5 (4)
C1—C6—C7—C8	−13.3 (4)	C9—C10—C15—C14	173.4 (3)

### Hydrogen-bond geometry (Å, °)

**Table:**

*D*—H···*A*	*D*—H	H···*A*	*D*···*A*	*D*—H···*A*
N1—H1N···O2^i^	0.86	2.03	2.872 (3)	168
O3—H3O···O4	0.82	1.80	2.525 (3)	146
C2—H2···O1^ii^	0.93	2.54	3.279 (3)	136
C14—H14···O2^iii^	0.93	2.58	3.435 (3)	153
C15—H15···N1	0.93	2.54	3.009 (4)	112

Symmetry codes: (i) *x*+1, *y*, *z*; (ii) −*x*, −*y*+1, −*z*+1; (iii) −*x*, −*y*, −*z*+1.
